# From regulatory approval to clinical practice: real-world eligibility for anti-amyloid monoclonal antibodies in an Italian dementia-care network

**DOI:** 10.1016/j.tjpad.2026.100655

**Published:** 2026-08-13

**Authors:** Giovanna Zamboni, Manuela Tondelli, Giulia Vinceti, Chiara Gallingani, Zeno Bercini, Aurora Barbieri, Chiara Carbone, Najara Iacovino, Silvia Cossutti, Daniela Ballotta, Simone Salemme, Annalisa Chiari

**Affiliations:** a Department of Biomedical, Metabolic and Neural Sciences, University of Modena and Reggio Emilia, via Campi 287, 41122 Modena, Italy; b Neurology Unit, Azienda Ospedaliero-Universitaria di Modena, Via Pietro Giardini 1355, 41126 Modena, Italy; c International School of Advanced Studies, University of Camerino, Piazza Cavour 19/f, 62032, Camerino, Italy

**Keywords:** Alzheimer's disease, anti-amyloid monoclonal antibodies, real-world eligibility, appropriate use recommendations

To the Editor,

The European authorisation of lecanemab and donanemab has shifted the implementation debate from whether anti-amyloid monoclonal antibodies can be used in early Alzheimer’s disease (AD) to how eligible patients can be identified safely, equitably, and sustainably in routine services. In Europe the product labels exclude from treatment APOE ε4/ε4 homozygous patients [1,2]. Therefore, national appropriate use recommendations (AURs) have aligned to this restriction further specifying diagnostic, imaging, safety, and monitoring requirements [3,4]. However, no equivalent estimate is yet available for Italy under Italian AURs. Real-world treatment-ready eligibility estimates are therefore essential to inform public health planning, budget impact, and regional service organisation.

We retrospectively assessed treatment-ready eligibility for lecanemab and donanemab among all patients with at least one visit between January 1, 2024, and December 31, 2025, at the Cognitive Neurology Clinic of the Modena University Hospital, a tertiary centre embedded within the dementia-care network of the Modena province (about 700,000 inhabitants). Eligibility was assessed using a stepwise sequential approach applying the Italian AURs [4] and estimated across three nested denominators (Fig. 1): all patients attending our service (*service cohort*), patients with a clinical diagnosis of mild cognitive impairment (MCI) or mild AD dementia irrespective of subsequent biomarker status (*clinical cohort*), and patients with biomarker-confirmed AD (*clinical-biological AD cohort*).

**Fig. 1:**
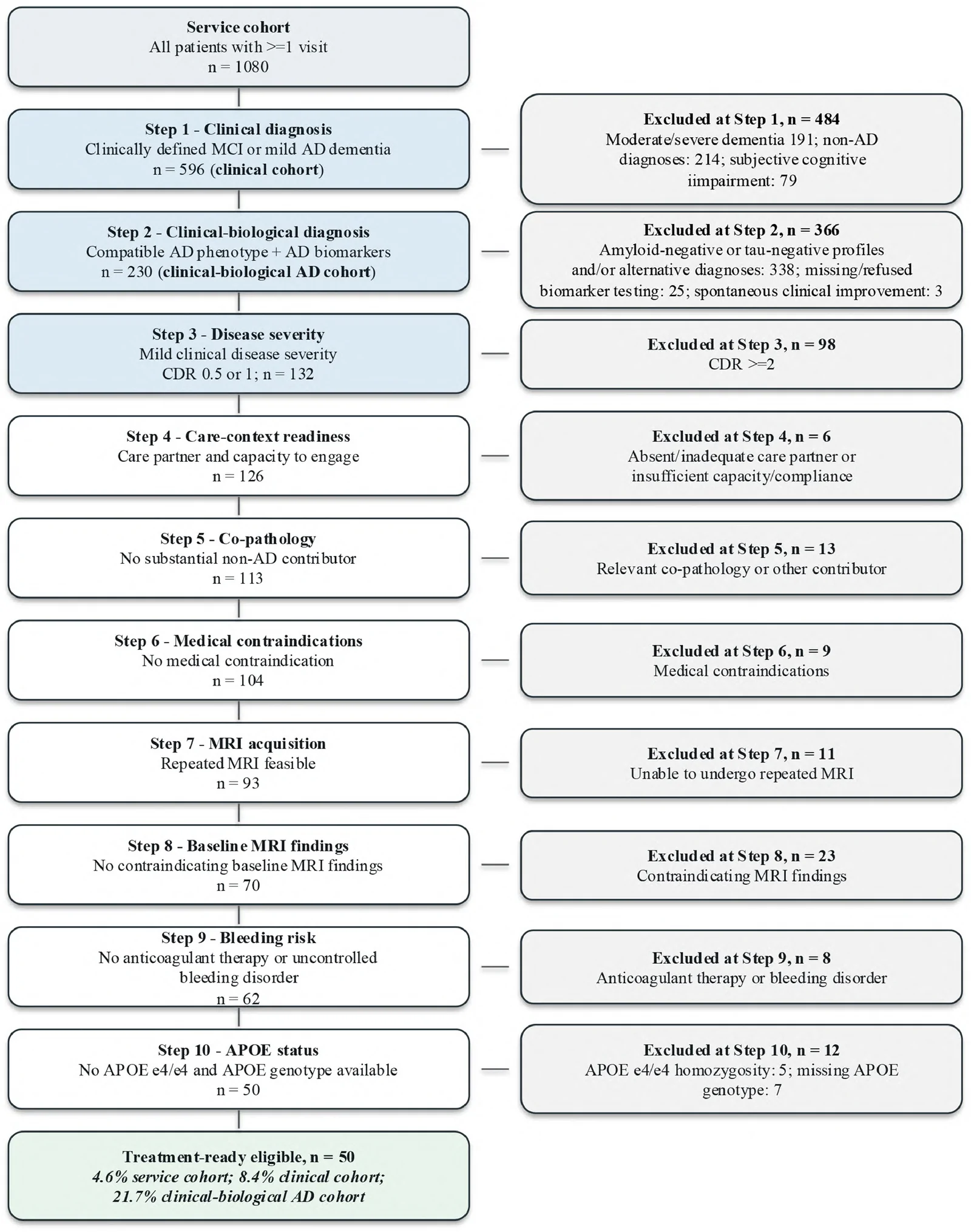
**Flowchart showing the sequential assessment of treatment-ready eligibility.** Sequential exclusions are mutually exclusive and assigned at the first ineligible step. AD = Alzheimer's disease; APOE = apolipoprotein E; AURs = appropriate use recommendations; CDR = Clinical Dementia Rating scale; EMA = European Medicines Agency; MCI = mild cognitive impairment; MRI = magnetic resonance imaging.

Among 1080 patients in the service cohort, 596 had MCI or mild AD dementia, and 230 had biomarker-confirmed clinical-biological AD. After applying all clinical, biological, disease-stage, care-context, magnetic resonance imaging (MRI), bleeding-risk, and APOE-related criteria, 50 patients were classified as treatment-ready. This corresponded to 4.6% of the service cohort, 8.4% of the clinical cohort, and 21.7% of the clinical-biological AD cohort. After biomarker testing, the largest attrition points were clinical stage (98 patients excluded because they were no longer within the mild clinical stage), MRI findings (23 excluded), and significant co-pathology or other contributors to cognitive impairment (13 excluded). Before APOE assessment, 62 patients fulfilled all previous eligibility requirements: five were excluded because of APOE ε4/ε4 homozygosity, seven because APOE genotype was unavailable, leaving 50 patients treatment-ready. In a criterion-specific attrition analysis on the clinical-biological AD cohort in which each post-biomarker criterion was evaluated independently of the others, the largest barriers after disease severity (98 excluded) were care-context requirements such as inadequate caregiver support, insufficient compliance, or lack of capacity to engage with treatment (58 excluded) and MRI-related criteria: 36 patients could not undergo MRI, and 54 of the remaining 194 patients had non-permissive findings on the MRI.

The main implementation message is that real-world eligibility is denominator-dependent (Table 1). A single percentage is potentially misleading unless the starting population is explicit. The 4.6% service-level estimate is relevant for referral planning and regional diagnostic capacity. The 8.4% estimate among patients with a clinical diagnosis of MCI or mild AD dementia is relevant for memory-clinic triage and biomarker demand. The 21.7% estimate among biomarker-confirmed patients is most relevant for prescribing, infusion capacity, MRI monitoring, and adverse-event preparedness.

**Table 1:** Denominator-specific eligibility for anti-amyloid monoclonal antibodies in the Modena dementia-care network.

Eligibility level	Definition	Numerator / denominator	Estimate	Main implementation meaning
**Service cohort**	All patients with ≥1 visit in 2024–2025	1080 / 1080	100%	Referral, triage, and service-level diagnostic workload
**Clinical cohort**	MCI or mild AD dementia	596 / 1080	55.2%	Population requiring diagnostic-pathway stratification
**Clinical-biological AD cohort**	Compatible syndrome plus AD biomarkers	230 / 596	38.6%	Biomarker-confirmed pool for treatment-readiness assessment
**Eligible before APOE assessment**	Fulfilled criteria up to MRI and bleeding-risk steps	62 / 1080	5.7%	Pre-genotyping treatment-readiness workload
**After excluding APOE ε4/ε4**	EMA-aligned APOE restriction applied	57 / 1080	5.3%	Potentially eligible under APOE restriction
**Conservative treatment-ready estimate**	APOE ε4/ε4 and missing APOE excluded	50 / 1080	4.6%	Final treatment-ready service-level estimate

Data are reported across sequential, nested denominators to distinguish service-level demand, diagnostic-pathway demand, biomarker-confirmed treatment-readiness assessment, and final conservative treatment-ready eligibility. Percentages are calculated using the denominator shown in each row unless otherwise specified. **AD** = Alzheimer’s disease; **APOE** = apolipoprotein E; **EMA** = European Medicines Agency; **MCI** = mild cognitive impairment; **MRI** = magnetic resonance imaging.

Post-biomarker attrition was not driven only by biological confirmation or APOE status. In the clinical-biological AD cohort, disease severity was the largest sequential barrier. MRI-related exclusions, relevant co-pathology, medical and concomitant drugs contraindications or frailty, and care-context readiness further reduced treatment readiness. In a criterion-specific analysis, care-context requirements were also frequent, highlighting that treatment readiness depends not only on amyloid status and MRI safety but also on the presence of supportive family and social environment capable of sustaining repeated infusions, monitoring, and risk communication.

These findings have three implications. First, implementation planning should avoid estimating demand directly from epidemiological prevalence of MCI or mild AD dementia, because such estimates may overstate treatable numbers. Second, eligibility assessment should begin upstream, before advanced biomarker testing whenever possible, through structured evaluation of clinical stage, major contraindications, MRI feasibility, care-partner availability, and capacity to engage with monitoring. Third, eligibility should be communicated to patients and caregivers as a layered judgement, not as an automatic entitlement to treatment or, conversely, as a withdrawal of care when criteria are not met.

This was the first real-world Italian eligibility study to apply the APOE ε4/ε4 restriction, to provide simultaneous estimates across three complementary denominators, and to be conducted in a centre embedded in a provincial network. Our findings indicate that only 4.6% of all patients seen in a tertiary cognitive neurology clinic were treatment-ready, similar to Swedish real-world data [5].

For Italy and comparable European health systems, anti-amyloid monoclonal antibody implementation will require explicit denominator-based planning, equitable access to biomarker confirmation and MRI monitoring, transparent APOE genotyping pathways, and safeguards against excluding patients through poorly governed referral or triage processes. The central challenge is therefore not only how many patients will receive treatment, but how many must pass through a structured diagnostic pathway to identify the small subset who are genuinely treatment-ready.

## Ethics approval

The study was approved by the local Ethics Committee as part of Protocol No. 372/2021, approved on Nov 2, 2021. Because this was a retrospective review of routinely collected data, no additional study-specific clinical assessments were performed.

## Data statement

Individual participant data cannot be made openly available because they contain sensitive health information and, even after pseudonymisation, may remain personal data under the EU General Data Protection Regulation. Deidentified aggregate data and analytic code may be made available upon reasonable request to the corresponding author, subject to approval by the local data protection officer where required.

## Declaration of generative AI and AI-assisted technologies

During preparation of this manuscript, the authors used UNIMORE-approved modalities of ChatGPT to support language editing and drafting of non-substantive text. The authors reviewed and edited all AI-assisted output, verified all factual content, analyses, and references, and take full responsibility for the final content of the manuscript. No patient-identifiable information was entered into the AI tool.

## Funding

This study received no specific external funding.

## CRediT authorship contribution statement

**Giovanna Zamboni:** Conceptualization, Data curation, Formal analysis, Investigation, Methodology, Project administration, Writing – original draft, Writing – review & editing. **Manuela Tondelli:** Conceptualization, Data curation, Formal analysis, Methodology, Writing – original draft, Writing – review & editing. **Giulia Vinceti:** Conceptualization, Data curation, Formal analysis, Investigation, Methodology, Writing – review & editing. **Chiara Gallingani:** Conceptualization, Data curation, Formal analysis, Investigation, Methodology, Writing – review & editing. **Zeno Bercini:** Conceptualization, Data curation, Formal analysis, Methodology, Writing – review & editing. **Aurora Barbieri:** Conceptualization, Data curation, Formal analysis, Writing – review & editing. **Chiara Carbone:** Conceptualization, Data curation, Formal analysis. **Najara Iacovino:** Conceptualization, Data curation, Formal analysis. **Silvia Cossutti:** Conceptualization, Data curation, Formal analysis. **Daniela Ballotta:** Conceptualization, Data curation, Formal analysis. **Simone Salemme:** Conceptualization, Data curation, Formal analysis, Investigation, Methodology, Writing – original draft, Writing – review & editing. **Annalisa Chiari:** Conceptualization, Data curation, Formal analysis, Investigation, Methodology, Writing – original draft, Writing – review & editing.

## Declaration of competing interest

The authors declare that they have no known competing financial interests or personal relationships that could have appeared to influence the work reported in this paper.
